# Protease 2A induces stress granule formation during coxsackievirus B3 and enterovirus 71 infections

**DOI:** 10.1186/s12985-014-0192-1

**Published:** 2014-11-20

**Authors:** Shuo Wu, Yan Wang, Lexun Lin, Xiaoning Si, Tianying Wang, Xiaoyan Zhong, Lei Tong, Ying Luan, Yang Chen, Xiaoyu Li, Fengmin Zhang, Wenran Zhao, Zhaohua Zhong

**Affiliations:** Department of Microbiology, Harbin Medical University, Harbin, 150081 China; Department of Cell Biology, Harbin Medical University, Harbin, 150081 China; Division of Gastroenterology and Hepatology, Department of Medicine, University of Florida-Jacksonville, Jacksonville, FL 32206 USA

**Keywords:** Stress granule, 2A protease, Coxsackievirus B, Enterovirus 71

## Abstract

**Background:**

Stress granules (SGs) are granular aggregates in the cytoplasm that are formed under a variety of stress situations including viral infection. Previous studies indicate that poliovirus, a member of *Picornaviridae*, can induce SG formation. However, the exact mechanism by which the picornaviruses induce SG formation is unknown.

**Method:**

The localization of SG markers in cells infected with coxsackievirus B3 (CVB3) or enterovirus 71 (EV71) and in cells expressing each viral protein was determined via immunofluorescence assays or plasmid transfection. Eight plasmids expressing mutants of the 2A protease (2A^pro^) of CVB3 were generated using a site-directed mutagenesis strategy. The cleavage efficiencies of eIF4G by CVB3 2A^pro^ and its mutants were determined via western blotting assays.

**Results:**

In this study, we found that CVB3 infection induced SG formation, as evidenced by the co-localization of some accepted SG markers in viral infection-induced granules. Furthermore, we identified that 2A^pro^ of CVB3 was the key viral component that triggered SG formation. A 2A^pro^ mutant with the G122E mutation, which exhibited very low cleavage efficiency toward eIF4G, significantly attenuated its capacity for SG induction, indicating that the protease activity was required for 2A^pro^ to initiate SG formation. Finally, we observed that SGs also formed in EV71-infected cells. Expression of EV71 2A^pro^ alone was also sufficient to cause SG formation.

**Conclusion:**

Both CVB3 and EV71 infections can induce SG formation, and 2A^pro^ plays a crucial role in the induction of SG formation during these infections. This finding may help us to better understand how picornaviruses initiate the SG response.

**Electronic supplementary material:**

The online version of this article (doi:10.1186/s12985-014-0192-1) contains supplementary material, which is available to authorized users.

## Background

Stress granules (SGs) are granular aggregates formed in the cytoplasm of eukaryotic cells exposed to a variety of environmental stress conditions, e.g., heat shock, UV irradiation, hypoxia, endoplasmic reticulum stress, and viral infection [1,2]. SGs typically contain translationally silent mRNAs, 40S ribosomal subunits, eukaryotic initiation factors (eIFs) such as eIF4E, eIF4G, eIF4A, eIF4B, eIF3, and eIF2, and RNA-binding proteins (RBPs), including poly(A)-binding protein (PABP1), the embryonic lethal abnormal vision (ELAV) Hu protein (HuR), polysomal ribonuclease 1 (PMR-1), tristetraprolin (TTP), T-cell-restricted intracellular antigen 1 (TIA1), TIA-1-related protein (TIAR), fragile X mental retardation protein (FMRP), and Ras-Gap SH3-binding protein (G3BP1) [1,2]. Among these recruited proteins, HuR, TIA1, and G3BP1 can act as markers of SGs [1–4].

Numerous viruses have been shown to interact with SGs with different effects [5–7]. Some viruses induce stable SG formation during infection, e.g., respiratory syncytial virus (RSV). Some viruses, e.g., mammalian orthoreoviruses (MRV), Semliki Forest viruses (SFV), and hepatitis C viruses (HCV) have been shown to induce SG formation but to disassemble these granules as infection proceeds. Poliovirus (PV) induces SG formation early during infection and later inhibits SGs through the viral 3C^pro^-mediated cleavage of G3BP1. Meanwhile, TIA1-containing foci devoid of other SG-defining components, such as initiation factors and most mRNAs were observed late post-infection. Some viruses, e.g., West Nile virus (WNV), dengue virus (DV), rhesus rotavirus (RRV), type 1 human T-cell leukemia viruses (HTLV-1), and human immunodeficiency viruses (HIV) were found to be able to suppress SG formation [5–7]. Cardioviruses with mutant leader (L) proteins, such as Theiler’s murine encephalomyelitis virus (TMEV), encephalomyocarditis virus (EMCV) and Saffold virus (SAFV) induce SG formation throughout the infection, while expression of the L protein during infection efficiently blocks SG formation. Similarly, influenza A virus (IAV) infection fails to induce SGs unless viruses with NS1 mutations are used [5–9]. In some cases, SGs play an important role in host antiviral defense, e.g., IAV, MRV and vaccinia virus (VV). However, SGs induced by RSV can promote virus replication [5–7].

Group B coxsackieviruses (CVBs) are the major pathogens of human viral myocarditis and dilated cardiomyopathy [10,11]. Enterovirus 71 (EV71) is the major pathogen of human hand, foot, and mouth disease (HFMD) [12]. Both CVBs and EV71 belong to the enterovirus genus of the *Picornaviridae* family. The picornavirus genome is a ~7.0-kb-to-8.5-kb single-stranded positive-sense RNA (+ssRNA) that is composed of a single open reading frame (ORF) and two untranslated regions (UTRs) at its 5′ and 3′ flanks. The genome can act as an mRNA encoding a polyprotein that is proteolytically processed by viral proteinases into structural and nonstructural proteins [13]. Picornaviruses induce multiple alterations in host cells to facilitate its replication. Suppressing cellular biosynthesis by viral proteinases 2A (2A^pro^) and 3C (3C^pro^) is one of the most notable alterations [13–17]. Upon the cleavage of eIF4G, eIF5B, and PABP by 3C^pro^ and 2A^pro^, picornaviruses can shut off cap-dependent translation and terminate cellular biosynthesis [13–17]. In addition, through the cleavage of nuclear pore complex proteins (Nups), 2A^pro^ can alter RNA and protein trafficking between the nucleus and cytoplasm [13]. A recent report shows that 2A^pro^ of PV, a member of *Picornaviridae*, can cause a dramatic nuclear-cytoplasm re-localization of SRp20, a cellular splicing factor that is also defined as an IRES trans-acting factor (ITAF) [18].

Recent studies indicate that PV induces unique SGs in infected cells [19–21]. The PV-induced SGs contain certain components that do not localize to SGs induced by oxidative stress, e.g., Sam68 and SRp20 [20,22]. However, the exact mechanism by which the picornavirus induces SG formation is unknown. In this study, we confirmed that CVB type 3 (CVB3) and EV71 also induce SG formation. We found that 2A^pro^ plays a crucial role in SG induction because 2A^pro^ alone is sufficient to trigger SG formation. Our findings may help us to better understand the mechanism by which picornaviruses initiate SG formation.

## Results

### CVB3 infection induces SG formation

**Figure 1:**
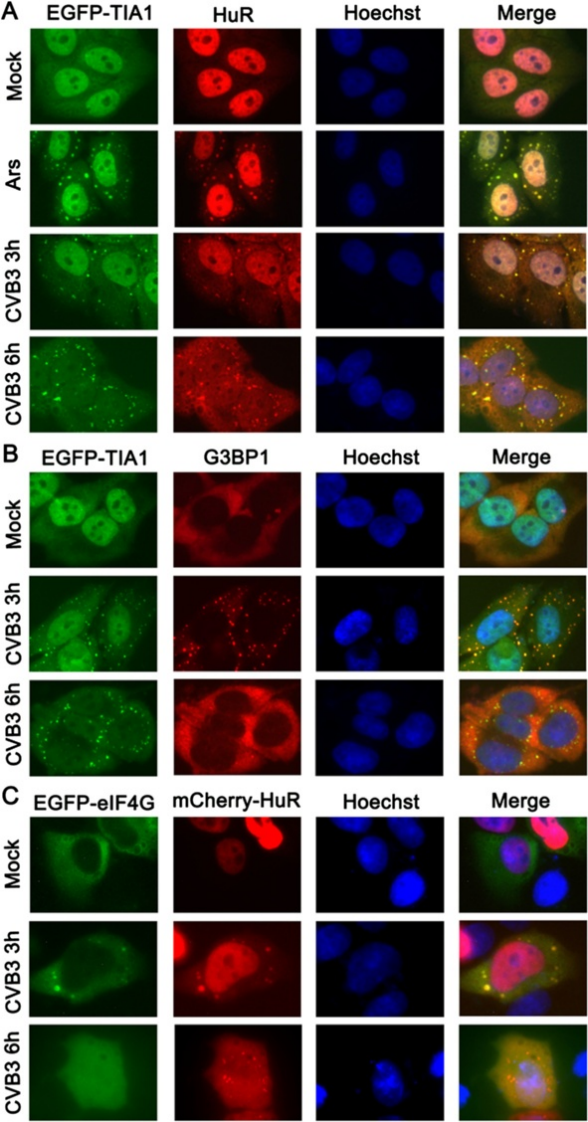
**CVB3 infection induces SG formation. (A)** HeLa^EGFP-TIA1^ cells were mock infected, exposed to 0.5 mM NaArs for 30 min, or infected with CVB3 (MOI =10) for 3 h. The cells were then fixed and stained for HuR. **(B)** HeLa^EGFP-TIA1^ cells were infected with CVB3 as discribed in **(A)**. The cells were then fixed and stained for G3BP1. **(C)** HeLa cells, co-transfected with pEGFP-eIF4G and pmCherry-HuR, were infected with CVB3 as discribed in **(A)**. The cells were then fixed and imaged. Nuclei were identified by Hoechst 33342 staining. Cells were examined using a fluorescence microscopy (×400).

To facilitate observing SG formation during CVB3 infection, we constructed a HeLa cell line (HeLa^EGFP-TIA1^), constitutively expressing EGFP-TIA1. SG formation was first determined by observing the expression and localization of TIA1 and HuR. HeLa^EGFP-TIA1^ cells were infected with CVB3 (MOI = 10) or treated with 0.5 mM NaArs for 30 min. In the mock-treated cells, EGFP-TIA1 was distributed in both the cytoplasm and nucleus. In the Ars-treated and CVB3-infected cells, EGFP-TIA1 was granularly distributed in the cytoplasm. The granules began to emerge in the cytoplasm of the CVB3-infected cells at 3 h post-infection (p.i.) (Figure 1).

Using an anti-HuR or anti-G3BP1 antibody, we found that HuR was predominantly localized in the nucleus of the mock-infected cells, but it re-localized to the cytoplasmic granules after both Ars treatment and CVB3 infection (Figure 1A). G3BP1 was distributed evenly in the cytoplasm of the mock-infected cells but formed granules in the CVB3-infected cells at 3 h p.i. but the G3BP1-positive granules dispersed at 6 h p.i. (Figure 1B). EGFP-TIA1 and HuR were co-localized in the granules in Ars-treated and CVB3-infected cells (Figure 1A). G3BP1 was also co-localized with EGFP-TIA1 in the granules of Ars-treated (data not shown) and CVB3-infected cells (Figure 1B).

Furthermore, in HeLa cells co-transfected with pEGFP-eIF4G and pmCherry-HuR, both mCherry-HuR and EGFP-eIF4G re-located from the nucleus to the cytoplasmic granules after CVB3 infection (MOI = 10) for 3 h (Figure 1C). We also noticed that the SGs contained HuR but no eIF4G at 6 h p.i. (Figure 1C). These data demonstrate that CVB3 induced typical SGs.

### 2A^pro^ is the key protein that triggers SG formation

**Figure 2:**
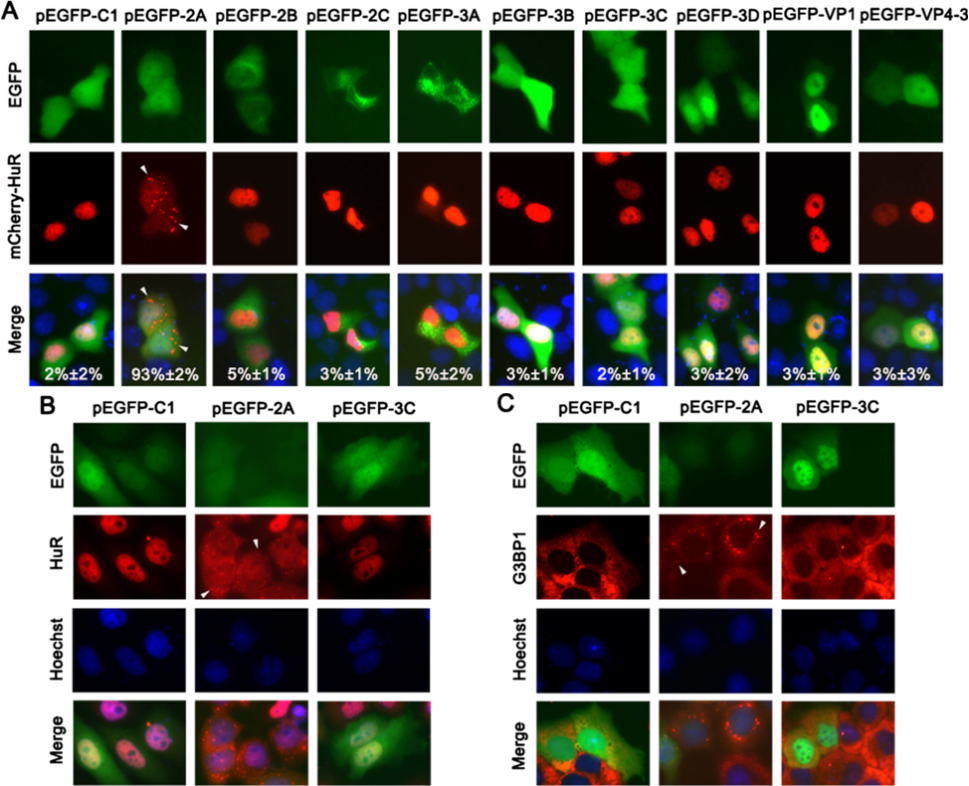
**CVB3 2A** ^**pro**^ **is identified as the viral component that induces SG formation in HeLa cells. (A)** HeLa cells were co-transfected with the pmCherry-HuR plasmid and one of the following plasmids: pEGFP-2A, pEGFP-2B, pEGFP-2C, pEGFP-3A, pEGFP-3B, pEGFP-3C, pEGFP-3D, pEGFP-VP1, or pEGFP-VP4-3. At 24 h post-transfection, the cells were imaged. The quantification of SG formation for each group was determined by analyzing the percentage of EGFP-expressing cells that contained three or more granules. At least 100 cells were counted for each experiment. The average of three independent experiments is indicated on the lower part of each panel. **(B)** HeLa cells were transfected with pEGFP-C1, pEGFP-2A, or pEGFP-3C. The cells were then fixed at 24 h post-transfection and stained for HuR via an anti-HuR rabbit polyclonal antibody and a CF555-labeled goat anti-rabbit secondary antibody. **(C)** HeLa cells were treated as discribed in **(B)** and then stained for G3BP1 via an anti-G3BP1 mouse monoclonal antibody and a CF555-labeled goat anti-mouse secondary antibody. Hoechst 33342 was used to counterstain the nuclei. The SGs were determined by observing the granular distribution of mCherry-HuR **(A)**, HuR **(B)**, and G3BP1 **(C)** in the cytoplasm under a fluorescence microscope (×400). The cytoplasmic granules are indicated by arrow heads.

To identify the viral component that triggered SG formation during CVB3 infection, HeLa cells were co-transfected with pmCherry-HuR and one of the nine plasmids expressing EGFP-tagged viral proteins (VP1, VP4-3, 2A^pro^, 2B, 2C, 3A, 3B, 3C^pro^, and 3D). We observed that mCherry-HuR was translocated from the nucleus to the cytoplasmic granules in cells transfected with pEGFP-2A after 24 h post-transfection (Figure 2A). We observed a similar result in Vero cells (Additional file 1: Figure S1). The nuclear localization of mCherry-HuR was not affected in cells expressing EGFP, VP1, VP4-3, 2B, 2C, 3A, 3B, 3C^pro^, or 3D (Figure 2A). Quantification of these experiments indicated that 93% (±2% [standard deviation]) of CVB3 2A^pro^-expressing cells exhibited SGs, while the percentages of SGs in other groups of cells did not exceed 5%.

By monitoring the endogenous HuR distribution using the anti-HuR antibody, a similar result was observed in cells only transfected with the viral protein-expressing plasmids (Figure 2B). Identically, G3BP1 detection showed that G3BP1 was distributed in cytoplasmic granules only in the cells expressing 2A^pro^ (Figure 2C). These results imply that 2A^pro^ is the key viral component that triggers SG formation during CVB3 infection.

### The protease activity is necessary for 2A^pro^ to trigger SG formation

**Figure 3:**
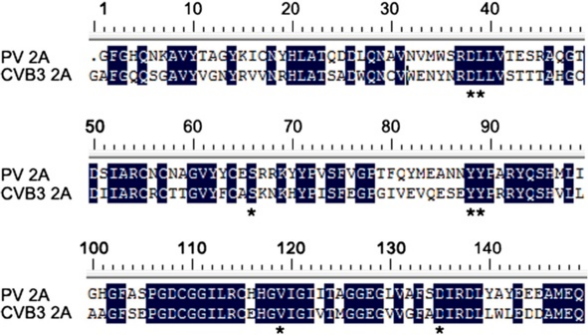
**Amino acid alignment of 2A** ^**pro**^ **in PV and CVB3.** Identical residues are in white lettering on a black background. The residues marked with asterisks (*) represent the amino acids of CVB3 2A^pro^ that would be mutated according to Additional file 1: Table S4.

**Figure 4:**
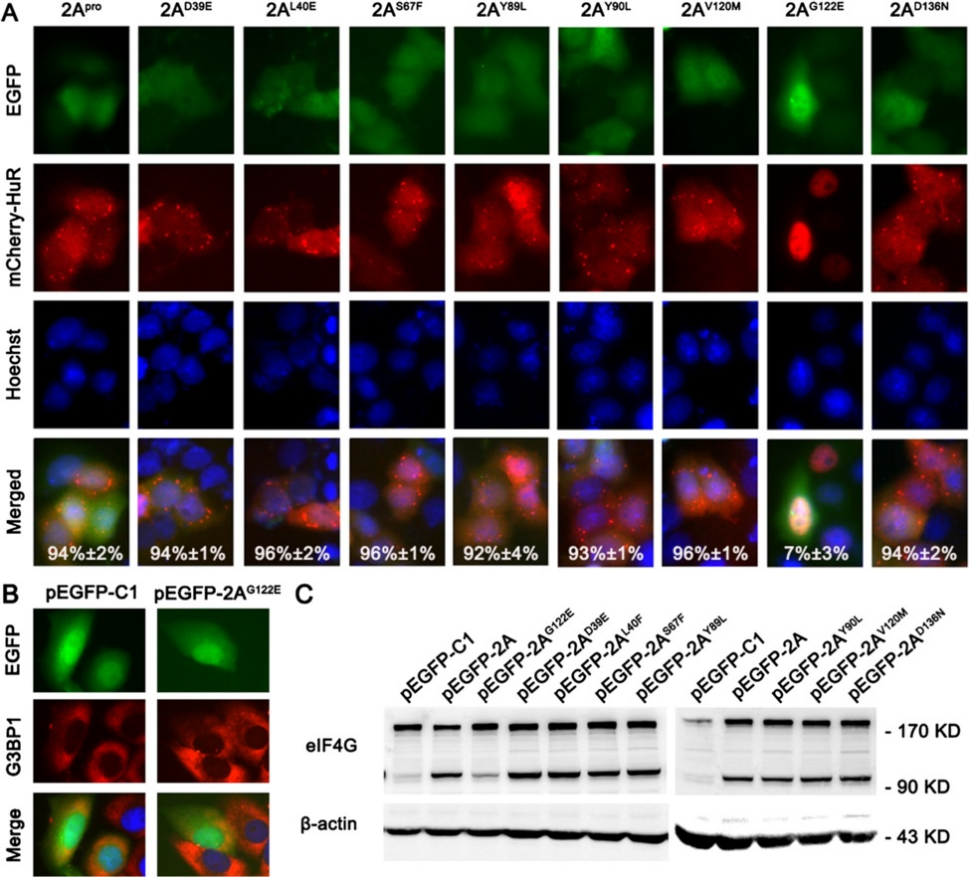
**The protease activity is required for 2A** ^**pro**^ **to induce SGs. (A)** HeLa cells were co-transfected with pmCherry-HuR and one of the plasmids (pEGFP-2A^D39E^, pEGFP-2A^L40F^, pEGFP-2A^S67F^, pEGFP-2A^Y89L^, pEGFP-2A^Y90L^, pEGFP-2A^V120M^, pEGFP-2A^G122E^, or pEGFP-2A^D136N^). The cells were observed at 24 h post-transfection. The percentage of EGFP-expressing cells with granules was quantified as in Figure 2A. The average of three independent experiments is indicated on the lower right of each panel. **(B)** HeLa cells were transfected with pEGFP-C1, or pEGFP-2A^G122E^. The cells were fixed at 24 h post-transfection and then stained for G3BP1. Nuclei were identified by Hoechst 33342 staining. The localization of the mCherry-HuR **(A)** or G3BP1 **(B)** proteins was determined using a fluorescence microscope (×400). **(C)** The protease activity of the 2A^pro^ mutants based on the cleavage efficiency of eIF4G was determined. HeLa cells were transfected with these 2A^pro^ mutant-expressing plasmids and the integrity of eIF4G was detected by western blotting at 24 h post-transfection.

Because the amino acid sequences of CVB3 2A^pro^ and PV 2A^pro^ were highly homologous (Figure 3), eight EGFP-tagged CVB3 2A^pro^ mutants including D39E, L40F, S67F, Y89L, Y90L, V120M, G122E, and D136N (marked with asterisks in Figure 3) were generated according to the PV 2A^pro^ variants that exhibited different cleavage activity toward eIF4G (Additional file 1: Table S4). Their protease activities, as indicated by their efficiency in cleaving eIF4G [23–25], were evaluated. Table 1 shows the results of their assessment of eIF4G cleavage activity. In cells expressing these mutants, only 2A^G122E^ showed attenuated protease activity compared with the prototype (Figure 4C and Table 1). The mutants including L40F, S67F, V120M, G122E, and D136N behaved as expected [24,25], while the mutants D39E, Y89L, and Y90L did not appear to behave as expected [23].

**Table 1:** **The eIF4G cleavage activity obtained with different variants of CVB3 2A** ^**pro**^

**2A mutant**	eIF4G cleavage activity*
Wild-type	+ + +
D39E	+ + +
L40F	+ + +
S67F	+ +
Y89L	+ + +
Y90L	+ +
V120M	+ + +
G122E	+/−
D136N	+ + +

*The 2A protease activity was determined to be undetectable (−), detectable in some assays (+/−), or positive (+ to + + +).

To determine whether the proteinase activity of 2A^pro^ is required for SG formation, HeLa cells were co-transfected with pmCherry-HuR and each of the mutant-expressing plasmids. We found that only a few SGs were formed in cells expressing 2A^G122E^. In contrast, there were abundant SGs formed in the cells expressing other 2A^pro^ mutants (Figure 4A). Quantitative analysis of these data revealed that only 7% (±3%) of EGFP-2A^G122E^-expressing cells contained SGs and over 90% of cells expressing the other 2A mutants remained positive for SGs. Similar results were observed for G3BP1 as the SG marker (Figure 4B). Therefore, we concluded that the protease activity was required for 2A^pro^ to induce SGs.

### EV71 and its 2A^pro^ also induce SG formation

**Figure 5:**
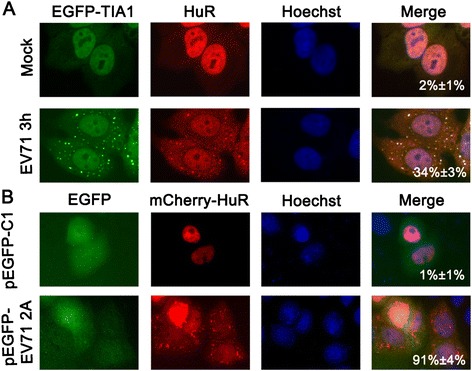
**EV71 and its 2A** ^**pro**^ **also induce SG formation. (A)** HeLa^EGFP-TIA1^ cells were mock-infected, or infected with EV71 (MOI =10) for 3 h. After fixation, the cells were stained for HuR. The quantification of SG formation was determined by analyzing more than 100 cells that contained three or more granules in multiple fields for each experiment. The average of three independent experiments is indicated on the lower right of each panel. **(B)** HeLa cells were co-transfected with pmCherry-HuR and pEGFP-EV71 2A or pEGFP-C1 and the cells were fixed at 24 h post-transfection and then imaged. Nuclei were stained with Hoechst 33342. The localization of EGFP-TIA1 **(A)**, HuR **(A)**, or mCherry-HuR **(B)** was determined under a fluorescence microscope (×400). The percentage of green cells possessing SGs was quantified as in Figure 2A.

We next examined whether SGs formed in cells infected with EV71. HeLa^EGFP-TIA1^ cells were mock-infected or infected with EV71 at an MOI of 10. In the infected cells, both EGFP-TIA1 and HuR underwent redistribution and co-localized to the cytoplasmic granules (Figure 5A). At 3 h p.i., approximately 34% of the infected cells showed granules. The granules also contained G3BP1 and eIF4G (data not shown), indicating that EV71 infection also induced SG formation. Similar to the experiments described in the previous section, HeLa cells were also co-transfected with pmCherry-HuR and pEGFP-C1 or pEGFP-EV71 2A. We found that 91% (±4%) of EV71 2A^pro^-expressed cells were positive for SGs (Figure 5B). A similar result was obtained in the infected Vero cells (Additional file 1: Figure S1). Thus, EV71 2A^pro^ can function in SG formation.

## Discussion

The interaction between viruses and host cells is critical for understanding the pathogenesis of viral infections. SG formation is one of the remarkable cellular events when cells sense an unfavorable stimulation [1,2]. Multiple viruses interact with SGs in different ways [5–7]. It has been reported that PV can induce SG formation. CVB3, EV71, and PV are members of the genus Enterovirus; however, they cause completely different types of diseases [10,12,26]. In this study, we found that CVB3 and EV71 could efficiently induce SG formation. The 2A^pro^ of both viruses were identified as the key for triggering SG formation. Our findings may help us to better understand the mechanism by which the picornaviruses initiate SG formation.

According to previous studies, TIA1, HuR, G3BP1, and eIF4G were chosen as the SG markers for our observations. TIA1 and HuR have been shown to be constantly recruited to SGs during PV infection, while G3BP1 and eIF4G can be detected in SGs at the early stage of the PV infection, but not later [19–21]. To facilitate observing the effect of virus on SG formation, we first constructed a HeLa^EGFP-TIA1^ cell line constitutively expressing TIA1. EGFP-TIA1 was distributed in cytoplasmic granules in CVB3- and EV71-infected HeLa^EGFP-TIA1^ cells (Figures 1 and 4). The granules were further confirmed as SGs because HuR, G3BP1, and eIF4G were also found in the granules (Figure 1). The SGs emerged as early as 3 h after the cells were infected with CVB3 or EV71 (MOI = 10) (Figures 1 and 5), suggesting that CVB3 and EV71 may efficiently induce SG formation. We also observed that G3BP1 and eIF4G were not localized to the SGs at the late stages of CVB3 or EV71 infection (data not shown), which was similar to the results during PV infection [19,21]. Thus, it is likely that SG formation may be a common feature in the response to picornavirus infections.

It would be interesting to know how picornaviruses initiate SG formation. SG formation may be triggered directly by a single viral component, or it could be indirectly induced by the distorted cellular environment when the picornavirus infections occur. Upon observation of the cellular response to every CVB3 protein, SGs traced by mCherry-HuR or endogenous HuR could only be viewed in cells expressing 2A^pro^ (Figure 2). HuR dominantly localizes in the nucleus. It is known that 2A^pro^ can disturb the nucleus-cytoplasm trafficking of macromolecules through cleaving nuclear pore proteins [13]. Thus, the translocalization of HuR may be a consequence of the nucleus-cytoplasm trafficking malfunction caused by 2A^pro^. For this reason, we validated our observation using the cytoplasmic SG marker G3BP1. Interestingly, G3BP1-labeled SGs could also only be viewed in cells expressing 2A^pro^ (Figure 2).

To further verify the role of 2A^pro^ in SG formation, we generated eight 2A^pro^ mutants based on studies of PV 2A^pro^ [20,21,23]. Among them, the protease activity of 2A^G122E^ was attenuated shown by measuring the cleavage efficiency of eIF4G. Accordingly, 2A^G122E^ did not induce SG formation, indicating that the protease activity was required for 2A^pro^ to trigger SG formation (Figure 4). Our results suggest that 2A^pro^ may be the key viral protein of CVB3 and EV71 to induce SG formation.

The role of 2A^pro^ in SG formation may be similar in PV infection. A recent study showed that PV infection could cause the redistribution of SRp20, a nucleocytoplasmic shuttling splicing factor [18]. The ectopic expression of PV 2A^pro^ was sufficient to cause the re-localization of SRp20 from the nucleus to the cytoplasmic granules [18]. A later study demonstrated that SRp20 could co-localize with TIA1 in the cytoplasmic granules of PV-infected cells, suggesting that SRp20 might also be a SG component [22]. These observations provide extra evidence for the role of 2A^pro^ in SG formation during picornavirus infections.

In summary, our findings help us to better understand the mechanism by which picornaviruses initiate SG formation in infected cells. However, the cellular components that 2A^pro^ targets to trigger SG formation remain unknown. Further study is needed to identify these targets to elucidate this cellular event.

## Conclusion

In this study, we demonstrated that both CVB3 and EV71 infections can induce SG formation and that 2A^pro^ plays a crucial role in the induction of SG formation during these infections. The protease activity was necessary for 2A^pro^ to trigger SG formation. This novel finding may help us to better understand the mechanism by which picornaviruses initiate the SG response.

## Methods

### Cell lines and viruses

HeLa cells were grown in Dulbecco’s modified Eagle medium (DMEM) (Invitrogen, Carlsbad, CA) supplemented with 8% (growth medium) or 5% (maintaining medium) fetal bovine serum (FBS) (Biological Industries Israel). A HeLa cell line stably expressing EGFP-TIA1 was established as described previously [27]. HeLa cells were cultured in 12-well plates to approximately 60% confluence and transfected with the plasmid pEGFP-TIA1 using Lipofectamine 2000 (Invitrogen). Cells were then grown in medium containing G418 (500 μg/ml). We replaced the culture medium every 2 days until colonies were observed. We acquired the stable cell line HeLa^EGFP-TIA1^ through 8 weeks of selection. HeLa^EGFP-TIA1^ cells stably expressing EGFP-TIA1 were grown in DMEM containing 10% FBS and 500 μg/ml G418. The CVB3 Woodruff strain was passaged in HeLa cells and titered by plaque assays as described previously [28]. The EV71 BrCr strain was passaged in Vero cells and titrated using the 50% tissue culture infective dose (TCID50) assay as described in our previous research [29].

### Plasmid construction

The plasmid expressing EGFP-tagged TIA1 or eIF4G was constructed in our laboratory. First, the EGFP-coding sequence was obtained from pEGFP-N1 (Clontech, Mountain View, CA) by polymerase chain reaction (PCR). The pcDNA3.1 (Invitrogen) and EGFP amplicons were digested by *Nhe* I and *Hind* III and then ligated at 16°C overnight with T4 ligase. The obtained plasmid was designated as pEGFP-C1. TIA1 and eIF4G cDNAs were amplified by reverse transcription PCR (RT-PCR) from the RNA extracts prepared from HeLa cells using TRIzol reagents (Invitrogen) and then cloned into pEGFP-C1. The resulting plasmids were designated as pEGFP-TIA1 and pEGFP-eIF4G, respectively. Similarly, the pmCherry-HuR plasmid, encoding a fusion protein of HuR and red fluorescence protein mCherry, was constructed based on pmCherry-C1 (Clontech). The plasmids were confirmed by DNA sequencing. The primers for the amplifications are listed in Additional file 1: Table S1.

Nine plasmids expressing EGFP-tagged VP1, VP4-VP2-VP3, 2A^pro^, 2B, 2C, 3A, 3B, 3C^pro^, or 3D of CVB3 were constructed as described previously [28,30] and designated as pEGFP-VP1, pEGFP-VP4-3, pEGFP-2A, pEGFP-2B, pEGFP-2C, pEGFP-3A, pEGFP-3B, pEGFP-3C, and pEGFP-3D, respectively. The primers are listed in Additional file 1: Table S2 and Table S3.

### Site-directed mutagenesis

Eight mutants of CVB3 2A^pro^, including 2A^D39E^, 2A^L40F^, 2A^S67F^, 2A^Y89L^, 2A^Y90L^, 2A^V120M^, 2A^G122E^ and 2A^D136N^, were generated by overlap PCR. Briefly, to generate 2A^D39E^, the pEGFP-2A DNA was amplified with 2A sense primer and 2A^D39E^ antisense primer, and with 2A^D39E^ sense primer and 2A antisense primer, respectively (Additional file 1: Table S3). The PCR products were purified and mixed together. The mixture was amplified with 2A sense and antisense primers. The resultant DNA was digested with *Hind* III and *Xba* I and inserted into the cloning site of pEGFP-C1. These plasmids were designated as pEGFP-2A^D39E^, pEGFP-2A^L40F^, pEGFP-2A^S67F^, pEGFP-2A^Y89L^, pEGFP-2A^Y90L^, pEGFP-2A^V120M^, pEGFP-2A^G122E^, and pEGFP-2A^D136N^, respectively. The digested fragments were mixed and ligated to pEGFP-C1/*Hind* III *+ Xba* I. All plasmids were confirmed by DNA sequencing.

### Transfection

For virus infection, HeLa cells were seeded in 24-well plates and grown to approximately 60% confluence. The cells were then co-transfected with 0.1 μg pmCherry-HuR and 0.5 μg pEGFP-C1, or pEGFP-eIF4G using 1 μl Lipofectamine 2000 (Invitrogen) per well. To express the viral proteins, cells were seeded in 24-well plates and grown to approximately 70% confluence. Cells were then co-transfected with 0.3 μg pmCherry-HuR and 0.3 μg plasmid encoding EGFP-tagged CVB3 2A^pro^, 2B, 2C, 3A, 3B, 3C^pro^, 3D, VP1, VP4-VP2-VP3, 2A^D39E^, 2A^L40F^, 2A^S67F^, 2A^Y89L^, 2A^Y90L^, 2A^V120M^, 2A^G122E^, or 2A^D136N^; EV71 2A^pro^; or the empty vector pEGFP-C1. Five hours later, the culture media were removed and replaced with fresh media. Images were taken using an Axiovert 200 fluorescence microscope (Carl Zeiss, Gottingen, Germany) at 24 h post-transfection. Some cells were seeded in 48-well plates and transfected with plasmid encoding EGFP-tagged CVB3 2A^pro^, 2A^G122E^ or 3C^pro^. Control cells were transfected with pEGFP-C1. After 24 h post-transfection, the cells were fixed for immunofluorescence assay of HuR and G3BP1. To investigate eIF4G cleavage, HeLa cells were transfected with plasmid encoding EGFP-tagged CVB3 2A^pro^, 2A^D39E^, 2A^L40F^, 2A^S67F^, 2A^Y89L^, 2A^Y90L^, 2A^V120M^, 2A^G122E^ or 2A^D136N^. Cells were harvested at 24 h post-transfection. Whole-cell lysates were subjected to sodium dodecyl sulfate-polyacrylamide gel electrophoresis (SDS-PAGE) and western blotting analysis.

### Virus infection

HeLa^EGFP-TIA1^ cells were mock-infected, or infected with CVB3 or EV71 at a multiplication of infection (MOI) of 10. At 3 h p.i., HeLa^EGFP-TIA1^ cells were fixed for immunofluorescence assays. HeLa cells, co-transfected with pmCherry-HuR and pEGFP-C1, or pEGFP-eIF4G or pEGFP-eIF4G^G681E^, were mock-infected or infected with CVB3 (MOI = 10). At 3, 4, or 6 h p.i., the cells were washed once with phosphate-buffered saline (PBS), and processed for imaging using an Axiovert 200 fluorescence microscope.

### Arsenite (Ars) treatment

Ars has been widely used as a stimulator of SGs [2]. To induce SGs, cells were treated with sodium arsenite (NaArs) (Sigma-Aldrich, St. Louis, MO) at a concentration of 0.5 mM in growth medium for 30 min.

### Immunofluorescence

Following virus infection, Ars treatment, or plasmid transfection as described above, the cells were washed once with PBS and then fixed with 4% paraformaldehyde at room temperature for 30 min. The cells were treated with 0.25% Triton X-100 for 10 min and blocked with PBS containing 1% bovine serum albumin (BSA) for 30 min at room temperature. Cells were then incubated overnight at 4°C with primary antibody diluted in blocking buffer and with secondary antibody for 2 h at room temperature in the dark. G3BP1 was detected via a monoclonal anti-G3BP1 antibody (611126, BD Transduction Labs, San Jose, CA) at a dilution of 1:200 and a CF555-labeled goat anti-mouse secondary antibody (20231, Biotium, Hayward, CA) at a dilution of 1:1000. HuR was detected via a polyclonal antibody (11910-1-AP, Proteintech Group Inc., Chicago, IL) at a dilution of 1:200 and a CF555-labeled anti-rabbit secondary antibody (20232, Biotium) at a dilution of 1:1000. The cells were then washed three times with PBS and stained with Hoechst 33342 (0.4 μg/ml in PBS). Images were taken using an Axiovert 200 fluorescence microscope.

### Western blotting

Proteins were extracted from the treated cells using Pierce RIPA Buffer with PMSF cocktail. Approximately 2 μg of the extracted proteins were applied to SDS-PAGE. The separated proteins were transferred to a polyvinylidene fluoride (PVDF) membrane (0.45 μm, Millipore, Billerica, MA), which was blocked with 5% nonfat milk for 2 h at 37°C and incubated with primary antibody overnight at 4°C. A polyclonal rabbit anti-eIF4G antibody (15704-1-AP, Proteintech Group Inc.) was 1:1000 diluted to detect eIF4G; a polyclonal anti-β-actin antibody (sc-130301, Santa Cruz Biotechnology, SantaCruz, CA) was 1:1000 diluted to detect β-actin. After a standard washing, the membrane was incubated with horse radish peroxidase (HRP)-labeled secondary antibody (Zhongshan Goldenbridge Biotech, Beijing, China) for 1 h at room temperature and washed again. The blots were stained using Super Signal kit (Pierce, Rockford, IL) and imaged by a LAS4000 charge-coupled camera (Fujifilm, Tokyo, Japan). The β-actin was employed as a loading control.

## Additional file

Additional file 1: Table S1. PCR primers for the construction of plasmids pEGFP-C1, pEGFP-TIA1, pmCherry-HuR , pEGFP-eIF4G and pEGFP-eIF4G^G681E^. **Table S2.** PCR primers for the construction of plasmids expressing CVB3 protein 2B, 3A, 3B, 3C, and 3D. **Table S3.** PCR primer sequences used for the site-directed mutagenesis of 2A^pro^. **Table S4.** The eIF4G cleavage activity obtained with different variants of PV 2A^pro^ [23–25]. **Figure S1.** Expression of CVB3 2A^pro^ or EV71 2A^pro^ in Vero cells results in SG formation. Vero cells were co-transfected with pmCherry-HuR and pEGFP-C1 or pEGFP-CVB3 2A or pEGFP-EV71 2A. Nuclei were identified by Hoechst 33342. The 2A expression and mCherry-HuR-positive SGs were determined using a fluorescence microscope (× 400) at 24 h post-transfection.
