# Supplementary material for: Protease 2A induces stress granule formation during coxsackievirus B3 and enterovirus 71 infections
Source: Virol J. 2014 Nov 20;11:192. doi: 10.1186/s12985-014-0192-1 (PMC4247557; doi:10.1186/s12985-014-0192-1)
Supplement: Additional file 1: Table S1. — PCR primers for the construction of plasmids pEGFP-C1, pEGFP-TIA1, pmCherry-HuR , pEGFP-eIF4G and pEGFP-eIF4GG681E. Table S2. PCR primers for the construction of plasmids expressing CVB3 protein 2B, 3A, 3B, 3C, and 3D. Table S3. PCR primer sequences used for the site-directed mutagenesis of 2Apro. Table S4. The eIF4G cleavage activity obtained with different variants of PV 2Apro [23–25]. Figure S1. Expression of CVB3 2Apro or EV71 2Apro in Vero cells results in SG formation. Vero cells were co-transfected with pmCherry-HuR and pEGFP-C1 or pEGFP-CVB3 2A or pEGFP-EV71 2A. Nuclei were identified by Hoechst 33342. The 2A expression and mCherry-HuR-positive SGs were determined using a fluorescence microscope (× 400) at 24 h post-transfection. [file 12985_2014_192_MOESM1_ESM.docx]

**Additional file**

Table S1. PCR primers for the construction of plasmids pEGFP-C1, pEGFP-TIA1, pmCherry-HuR , pEGFP-eIF4G and pEGFP-eIF4G^G681E^

| Target | Polarity | Sequences | Underlined |
| --- | --- | --- | --- |
| EGFP | Sense | ATATATGCTAGCATGGTGAGCAAGGGCGAGGAGCT | *Nde* I |
|  | Antisense | ATATATAAGCTTCGATCCGCCACCGCCAGAGCCACCTCCGCCTGA  ACCGCCTCCACCCTTGTACAGCTCGTCCATGCCG | *Hind* III |
| TIA1 | Sense | GCGAATAAGCTTGAGGACGAGATGCCCAAGACT | *Hind* III |
|  | Antisense | ATCGCGTCTAGATCACTGGGTTTCATACCCTGC | *Xba* I |
| HuR | Sense | ATATATGAATTCTGGTGGAGGCGGTTCAGGCGGAGGTGGCTCTGGCGGTGGCGGATCGTCTAATGGTTATGAAGA | *EcoR* I |
|  | Antisense | GCGCGCGGTACCTTATTTGTGGGACTTGTTGG | *Kpn* I |
| eIF4G | Sense  Antisense | ATATATAAGCTTAACAAAGCTCCACAGTCCACAGGCCCCCCA  ATGCGCTCTAGATCAGTTGTGGTCAGACTCCTCCTCTGCTTC | *Hind* III  *Xba* I |
| eIF4G^G681E^ | P1 | ATATATAAGCTTAACAAAGCTCCACAGTCCACAGGCCCCCCA | *Hind* III |
|  | P2 | GGCCCACCCCTTGGGGGCTCACGGGTGCTAAGGGTTGTC | *PflM* I |
|  | P3 | GACAACCCTTAGCACCCGTGAGCCCCCAAGGGGTGGGCC | *PflM* I |
|  | P4 | ATGCGCTCTAGATCAGTTGTGGTCAGACTCCTCCTCTGCTTC | *Xba* I |

Table S2. PCR primers for the construction of plasmids expressing CVB3 protein 2B, 3A, 3B, 3C, and 3D

| Protein | Polarity | Sequences | Underlined |
| --- | --- | --- | --- |
| 2B | Sense | ATATATAAGCTTGGTGGAGGCGGTTCAGGCGGAGGTGGCTCTGGCGGTGGCGGATCGGGAGTGAAGGACTATGTGGAAC | *Hind* III |
|  | Antisense | ATAGCGTCTAGATTATTGGCGTTCAGCCATGGGTATTCCGT | *Xba* I |
| 3A | Sense | ATATATAAGCTTGGTGGAGGCGGTTCAGGCGGAGGTGGCTCTGGCGGTGGCGGATCGGGTCCACCAGTATACAGAGAGAT | *Hind* III |
|  | Antisense | GCGCGTCTAGATTATTGAAAGCCTGCAAAGAGCTTGTATATTATAT | *Xba* I |
| 3B | Sense | ATATATAAGCTTGGTGGAGGCGGTTCAGGCGGAGGTGGCTCTGGCGGTGGCGGATCGGGTGCATATACAGGAATACCCAACC | *Hind* III |
|  | Antisense | GCGCGTCTAGATTACTGCACTTTTGCTTGCCTTAGGGTAGG | *Xba* I |
| 3C | Sense | ATATATAAGCTTGGTGGAGGCGGTTCAGGCGGAGGTGGCTCTGGCGGTG  GCGGATCGGGTCCTGCATTTGAATTTGCTGT | *Hind* III |
|  | Antisense | GCGCGTCTAGATTATTGTTCATCATTGAAATAGTGCT | *Xba* I |
| 3D | Sense | ATATATAAGCTTGGTGGAGGCGGTTCAGGCGGAGGTGGCTCTGGCGGTGGCGGATCGGGAGAGATCGAGTTTATTGAGAG | *Hind* III |
|  | Antisense | GCGCGTCTAGATTAGAAAGAGTCCAACCACTTC | *Xba* I |

Table S3. PCR primer sequences used for the site-directed mutagenesis of 2A^pro^

| Target | Polarity | Sequences (The mutated codons are underlined) | Bold italic |
| --- | --- | --- | --- |
| 2A^pro^ | Sense | ATATAT***AAGCTT***GGCGCATTTGGACAACAATCAGG | *Hind* III |
| 2A^pro^ | Antisense | GCGGCG***TCTAGA***TTACTGTTCCATTGCAT | *Xba* I |
| 2A^D39E^ | Antisense | GTCGTGCTCACTAAGAGCTCTCTGTTGTAATTTT |  |
| 2A^L40F^ | Antisense | GGTCGTGCTCACTAAGAAGTCTCTGTTGTAATTTTC |  |
| 2A^S67F^ | Antisense | GGTAGTGTTTGTTTTTGAACGCACAAAAGTACAC |  |
| 2A^Y89L^ | Antisense | GGTATCTCCTAGGGTATAACTCACTCTCTTGG |  |
| 2A^Y90L^ | Antisense | ATTGGTATCTCCTAGGTAAGTACTCACTCTCTTG |  |
| 2A^V120M^ | Antisense | GTCACAATGCCAATCATACCATGCTCACACC |  |
| 2A^G122E^ | Antisense | CCCCCCATGGTCACAATCTCAATGACACCATGCTC |  |
| 2A^D136N^ | Antisense | CAGGAGATCACGGATGTTTGCAAAGCCGACCAC |  |

Table S4. The eIF4G cleavage activity obtained with different variants of PV 2A^pro^ [22-24]

| 2A mutant | eIF4G cleavage activity* |  |
| --- | --- | --- |
| wild-type | + + + |  |
| D38E | - |  |
| L39F | + + | |
| S66F | + + + | |
| Y88L | +/- | |
| Y89L | +/- | |
| V119M | + + | |
| G121E | - | |
| D135N | + + + | |

*The 2A protease activity was determined to be undetectable (-), detectable in some assays (+/-), or positive (+ to + + +).


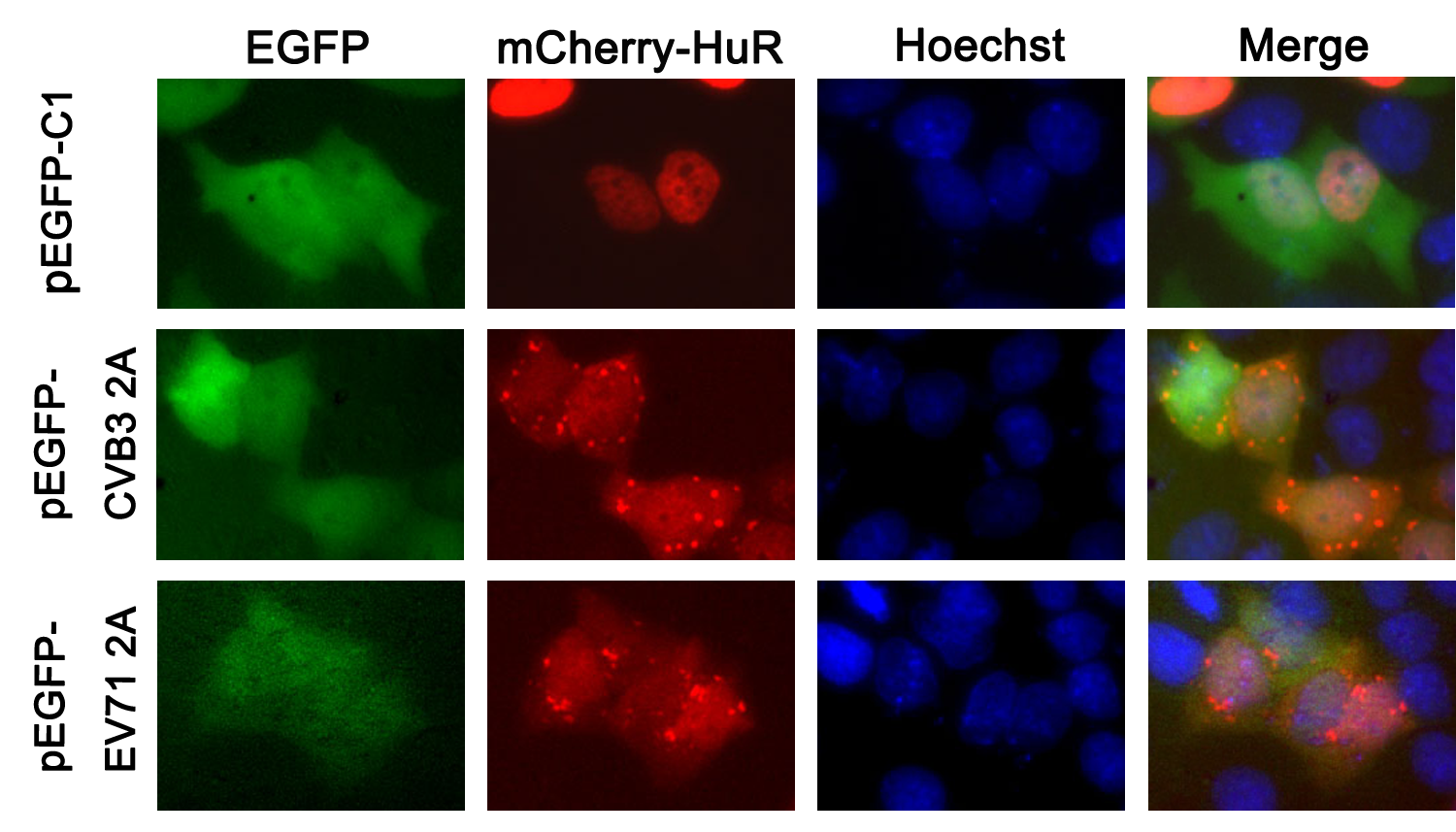


**Figure S1.** Expression of CVB3 2A^pro^ or EV71 2A^pro^ in Vero cells results in SG formation. Vero cells were co-transfected with pmCherry-HuR and pEGFP-C1 or pEGFP-CVB3 2A or pEGFP-EV71 2A. Nuclei were identiﬁed by Hoechst 33342. The 2A expression and mCherry-HuR-positive SGs were determined using a fluorescence microscope (× 400) at 24 h post-transfection.
